# The Nexus between Smart Sensors and the Bankruptcy Protection of SMEs

**DOI:** 10.3390/s22228671

**Published:** 2022-11-10

**Authors:** Pavol Durana, Katarina Valaskova

**Affiliations:** Department of Economics, Faculty of Operation and Economics of Transport and Communications, University of Zilina, Univerzitna 1, 010 26 Zilina, Slovakia

**Keywords:** bankruptcy, COVID-19 pandemic, earnings, Industry 4.0, small and medium-sized enterprises, smart sensors, Visegrad Four

## Abstract

Transportation, logistics, storage, and many other sectors provide a wide space for applying Industry 4.0. This era, with its components, represents the equipment necessary to obtain a unique competitive advantage. Being smart through sensors, big data, and digitalization corresponds not only to evolution but also provides protection for businesses in the face of depression. The COVID-19 pandemic caused collapses and defects for very large enterprises and large enterprises, especially for small and medium-sized enterprises (SMEs). This article focuses on SMEs and their profits from using smart sensors. Thus, the aim was to expose the striking effect of Industry 4.0 on earnings during the crisis in the Visegrad Four. The Mann–Kendall trend was used to map the consequences contrasting the period of 2016–2021. The investigation involved samples from 1221 Slovak, 259 Czech, 855 Polish, and 2156 Hungarian enterprises. The results showed that more than 80% of businesses did not have a negative trend in how their earnings changed over time. This fact was confirmed by a z-test for the comparison of one proportion for each analyzed country. The adaptation to Industry 4.0 strengthened the muscle for bankruptcy resilience during the crisis. In addition, it may encourage enterprises to be smart in the same or different sectors.

## 1. Introduction

Smart sensors are applied in sectors and their systems to indicate changes or monitor trends in controlling parameters during the Industry 4.0 era [1]. Small and medium-sized enterprises may determine and enhance their strategic position using them [2]. The COVID-19 pandemic caused a very turbulent and risky business environment, affecting all enterprises [3,4]. When an analytical solution to a particular examined problem is not possible or would be difficult to obtain, or when a comparison of analytical and model solutions is advised, models are used [5]. For the purpose of creating a model that will be used as a tool for future research, it is essential to gather as much information as possible from real-world solutions [6]. Thus, the consequences and impacts of a new crisis were hard to predict for all enterprises, especially in the area of SMEs [7,8]. The evidence of financial indebtedness and defaults in the V4 region was confirmed [9,10,11,12,13,14,15]. There have been occurrences of many approaches, behaviors, and intentions to maintain resilience and avoid bankruptcy during and after a given crisis [16,17,18,19,20]. New habits have been adopted and are now preferred [21,22].

Tijani et al. [23] advocated for enterprises to form strategic alliances and partnerships. Watson and Popescu [24] added the decisions leading to the acquisitions. Dempere [25] identified the factors of economic freedom that control the COVID-19 era. Javaid et al. [26] highlighted the importance of Industry 4.0 and its technologies in the fight against the coronavirus. Acioli et al. [27] identified Industry 4.0 as a facilitator in the erupting crisis. Its moderating role recognizes the conclusions by Narayanamurthy and Tortorella [28] or Hussain et al. [29]. Agrawal et al. [30] pointed out how important Industry 4.0 is and how important it will be in the future to solve problems such as COVID-19. Kubickova et al. [31] detected the acceleration in the enterprises to implement the tools of Industry 4.0 during the pandemic. Umair et al. [32] confirmed the catalyst due to the coronavirus and the significance of Industry 4.0 in the transportation sector as well. Those findings were based on reports by consulting companies and interactions with industry experts.

In general, Industry 4.0 is a fundamental requirement for process optimization, waste reduction, innovation introduction, and acquiring competitive advantages [33]. Its policies determine one of the crucial parameters of sustainable economic growth [34]. Lazanyi and Lambovska [35] tested V4 countries for the challenges and ways related to Industry 4.0 and concluded that national initiatives have been consistent. Based on this study, Slovakia, Czechia, Poland, and Hungary provide a great starting point to compare the similar effects of Industry 4.0 in SMEs. Habanik et al. [36] showed that the COVID-19 crisis has forced considerable application of Industry 4.0 in enterprises. The technological advances in smart sensors and systems, the interconnectivity of big data, automation, digitalization, and artificial intelligence have contributed to defining Industry 4.0 [37].

Thus, the aim of this research was to expose the striking effect of Industry 4.0 on earnings during the crisis in the Visegrad Four. Many studies have confirmed the dependence between the derivatives of Industry 4.0 and the maintenance of earnings for enterprises in general. The contribution of this study compared to previous studies is a comprehensive assessment of SMEs for the V4 region on the issue based on robust samples and confirmation of the significant effect of smart sensors against crises as evidenced by the COVID-19 pandemic example. The novelty consisted of using methodologies that combined trend analysis, heterogeneity occurrence, and the z-test. 

This article especially focuses on smart sensors; the last incentives have been as follows: Blake and Frajtova Michalikova [38] provided a quantitative literature review of ProQuest, Scopus, and the Web of Science for cognitive sensor networks; Nagy and Lazaroiu [39] focused on the techniques of sensor networks; Tucker [40] and Suler et al. [41] assessed sensor networks in cyber–physical system-based smart factories; and Townsend [42] and Harris [43] dealt with sensing technologies in planning and data-driven smart, sustainable cities.

Marinov et al. [44] showed that suitable “lightweight” algorithms are required for effective connectivity and smart control of the measurement processes in order to address the issues brought about by the resource limitations of smart devices. Blake et al. [45] added deep-learning-assisted smart process planning. Griffin and Krastev [46] extended smart process planning to transportation systems. Jiang and Qiu [47] delivered an investigation of multitarget localization and tracking. 

Valaskova et al. [48] linked systems of sensors for smart manufacturing. Bhargava et al. [49] monitored them in logistics and supply chain management. Ullo and Sinha [50] confirmed that smart sensors with advanced techniques such as artificial intelligence (AI) play an important role in other areas and sectors; e.g., agriculture. The significance of AI was highlighted in studies by Zhang and Wei [51] and Nica and Stehel [52] as well. Cheema et al. [53] emphasized the use of sensors to capture real-time parameters for smart farming. Novak et al. [54] and Adams and Krulicky [55] preferred to make decisions about products and manufacturing based on real-time sensor networks.

Ha et al. [56] enumerated the practical uses of sensing in the context of the two main categories of optical image sensing by describing the interconnection between the sensor technologies. Cohen [57] discussed interconnected sensor networks in digital urban governance. Sustainable urban governance networks were evaluated in the exploration of Evans and Horak [58]. Fonseca et al. [59] suggested improvements in quantitative traffic measurement toward smart city governance. Chapman [60] examined environmental sustainability by connecting sensors in cities.

Shirmohammadli and Bahreyni [61] prepared case studies that applied the information offered to address various real-world applications of smart sensors. Welch [62] focused on real-world applications for vehicles. Specifically, Nandutu et al. [63] reported the status of sensing in vehicle collisions. Mitchell [64] and Aldridge and Stehel [65] described the possibility of autonomous vehicle algorithms in transportation systems. However, Shen et al. [66] and Lewis [67] warned that many of the problems in this area depend on software systems, data quality, and verification.

Smart sensors lead to an increase in the accumulation of big data [68,69]. Gibson [70] comprehensively solved the analysis and management of big data. Dawson [71] determined the use of sensing big data in sustainable product lifecycle management. Aliahmadi et al. [72] concluded that thanks to smart sensors and the production of big data, managers can respond to conditions and act on changes.

Table 1 sums up the mentioned studies according to the solved area.

This article is structured as follows: A literature review of studies pertaining to the evaluation of smart sensors and the most recent incentives is highlighted first. The statistical techniques employed in the provided study are then demonstrated, together with the dataset that was used. The Section 3 includes the findings. In Section 4, the acquired results are contrasted with similar studies from the V4 region. In Section 5, the constraints of the study are summed up, and suggestions for future research are also made.

## 2. Materials and Methods

This study targeted SME enterprises. The choice was limited to businesses that were oriented toward smart sensors because this issue has been scarcely documented in the literature [73]. The period of investigation, 2016–2021, was purposely selected. The first part of the period allowed a focus before the COVID-19 crisis. Enterprises had to implement and use smart sensors until the beginning of the crisis; this covered the years from 2016 to 2019. The main aim was to expose the striking effect of Industry 4.0 on earnings during the crisis in the Visegrad Four. Thus, the second half of the time frame, from 2020 to 2021, was chosen to detect how smart sensors impacted the coronavirus era. The data was taken from the Orbis database, which was provided by Bureau van Dijk.

The earnings were chosen because owners, managers, or other external entities set the greatest momentousness for financial quantification [74,75]. It has been proven that earnings volatility or a rapid decrease in earnings may lead to bankruptcy [76,77,78]. The idea of bankruptcy protection involves the incentives that maintain the earnings measures at an appropriate level or eliminate the risk of a negative change in the earnings of the enterprises [79,80]. 

The financial situations of SMEs were expressed in this investigation as earnings before interest, tax, depreciation, and amortization (EBITDA). This indicator is recommended for use in the comparison of different sectors [81,82] because it eliminates the difference in economic results between different sectors as well as countries because it removes the impact of different tax policies and interest rates, especially the different depreciation and amortization standards.

The following methodology was used in this study:

1. *Creation of a sample.*

The outliers were not excluded because the sub-samples were not balanced to obtain a relevant and robust sample from V4, reflecting the actual situation in the sector [83,84,85,86]. Together, 4834 SMEs were related to Industry 4.0. However, the original Orbis database included the missing values for EBITDA. They were found and excluded, not replaced. Incomplete data were detected for 165 SMEs, 157 Czech SMEs, 18 Polish SMEs, and 3 Hungarian SMEs (Table 2). Table 2 also shows the final number of units included in this investigation. 

Each country was labeled with a number: 1 for Slovakia, 2 for Czechia, 3 for Poland, and 4 for Hungary. These numbers were used to name the countries in the tested hypotheses.

**Table 2:** Sampling of SMEs.

Number of SMEs	Slovakia (1)	Czechia (2)	Poland (3)	Hungary (4)
Original sample	1386	416	873	2159
Sample with incomplete data	165	157	18	3
Used sample	1221	259	855	2156

2. *Detection of a trend.*

There was a premise that smart sensors supported enterprises. Then, we could not reject the occurrence of no trend within EBITDA during 2016–2021. The existence of a monotonic trend was found by the Mann–Kendall trend test. The time series for Slovak, Czech, Polish, and Hungarian SMEs were checked for a negative trend against a distribution that was identical. 

Kliestik et al. [87] noted the null hypothesis was that the data came from a population with independent realizations and were identically distributed. There may be no trend. The alternative hypothesis was that the data followed a monotonic trend. This hypothesis can be tested for a positive, negative, or non-null trend. This test assessed the sign of the difference between later-measured data and earlier-measured data. The alternative hypothesis for the non-null did not test a specific trend. It confirmed only the occurrence of the trend. It may be formulated as follows: there exists either an upward or a downward monotonic trend.

The nonparametric statistic (*S*) is defined as:

$$S=\overset{n-1}{\underset{k=1}{\sum }}\overset{n}{\underset{j=k+1}{\sum }}\mathrm{sgn}\;\left(X_{j}-X_{k}\right)$$

with

$$\mathrm{sgn}\;\left(x\right)=\{\begin{array}{c} 1\\ 0\\ -1 \end{array}\;\begin{array}{c} \mathrm{if}\\ \mathrm{if}\\ \mathrm{if} \end{array}\;\begin{array}{c} x\\ x\\ x \end{array}\;\begin{array}{c} >\\ =\\ < \end{array}\;\begin{array}{c} 0\\ 0\\ 0 \end{array}$$

where $\mathrm{sgn}\left(X_{j}-X_{k}\right)$ is an indicator function that takes on the values 1, 0, or −1 according to the sign of $X_{j}-X_{k}.\;$ The mean of *S* is *E*[*S*] = 0 and the variance $\sigma^{2}$ is:

$$\sigma^{2}=\frac{1}{18}\;\left\{n\;\left(n-1\right)\;\left(2n+5\right)-\overset{p}{\underset{j=1}{\sum }}t_{j}\;\left(t_{j}-1\right)\;\left(2t_{j}+5\right)\right\}$$

where *p* is the number of the tied groups in the data set and *t_j_* is the number of data points in the *j*-th tied group. The statistic *S* is approximately normally distributed provided that the following *Z*-transformation is employed:

$$Z=\{\begin{array}{c} \frac{S-1}{\sigma }\\ 0\\ \frac{S+1}{\sigma } \end{array}\;\begin{array}{c} \mathrm{if}\\ \mathrm{if}\\ \mathrm{if} \end{array}\;\begin{array}{c} S\\ S\\ S \end{array}\;\begin{array}{c} >\\ =\\ < \end{array}\;\begin{array}{c} 0\\ 0\\ 0 \end{array}$$

where σ is the standard deviation. It is much easier to present statistics that are closely linked to *S* and more well-known, such as Kendall’s *τ,* which is given by:

$$\tau =\frac{S}{D}$$

where:

$$D={\left[\frac{1}{2}n\;\left(n-1\right)-\frac{1}{2}\overset{p}{\underset{j=1}{\sum }}t_{j}\;\left(t_{j}-1\right)\;\right]}^{\frac{1}{2}}\;{\left[\frac{1}{2}n\;\left(n-1\right)\;\right]}^{\frac{1}{2}}$$

Kendall’s *τ* is normalized; that is why it may take values from −1 to 1, with negative values detecting a downward trend and positive values an upward trend in a time series.

The following theory was developed and tested separately for each nation:

**H_a_.**  *Smart sensors did not balance earnings for Slovak (Czech, Polish, and Hungarian) SMEs. There was a negative trend confirmed for the period of 2016–2021.*

3. *Disclosure of a change*.

Pettitt’s test was run after the trend test for SMEs; it confirmed the negative development. It was done to trace heterogeneity and the year that changed the decrease in EBITDA as based on the study by Kanovsky [88]. There was a premise that pandemic years (2020 or 2021) affected the disruptions. The mentioned test defined whether EBITDA could be considered homogeneous within a time series or if there existed any year in which change was caused. This test was used with 100,000 Monte Carlo simulations to test the homogeneity because it is a nonparametric rank test that can reveal the single break point in continuous data [89].

Valaskova et al. [90] noted that the null hypothesis was that the T variables followed one or more distributions that had the same location parameter. The alternative hypothesis was that the year of change occurred. 

The nonparametric statistic is defined as:

$$K_{T}=\max\left|U_{t,T}\right|$$

where:

$$U_{t,T}=\overset{t}{\underset{i=1}{\sum }}\overset{T}{\underset{j=t+1}{\sum }}\mathrm{sgn}\left(X_{i}-X_{j}\right)$$

where the year of change of the time series is located at *K_T_* provided that the statistic is significant. The significance probability of *K_T_* is approximated for a *p*-value ≤ 0.05 as:

$$p≅2\exp\left(\frac{-6\;K_{t}^{2}}{T^{3}+T^{2}}\right)$$

The following theory was developed and tested separately for each nation:

**H_b_.**  *There was a phenomenon of heterogeneity in earnings for smart Slovak, Czech, Polish, and Hungarian SMEs. The pandemic years caused a negative disruption in the period of 2016–2021.*

4. *Confirmation of a proportion*.

The pandemic years were determined as the years of change within EBITDA. Then, the z-test for the comparison of one proportion was applied to confirm the percentage of SMEs that were protected during a crisis via smart sensors. The findings could be generalized after running this test based on the used samples.

Svabova et al. [91] noted that the null hypothesis was that the proportion of character π in the population was equal to the constant $\pi_{0}$ (a test proportion). The alternative hypothesis in this case was that the proportion of character π was greater than the chosen constant. The proportion occurred in the population at a higher rate than was assumed.

An assumption of the z-test is that the size of the sample must be sufficiently large while considering the occurrence of the required character within it. The fulfillment of this assumption is very important in order to be able to use the approximation of the distribution of the test statistics using the normal distribution. The sample is large enough if:

np(1−p)>9

where:

$$p=\frac{m}{n}$$

where m is the number of units with the required attributes (no trend in EBITDA) and n is the total range of the used sample.

The test statistic T is defined as:

$$T=\frac{p-\pi_{0}}{\sqrt{\frac{\pi_{0}\left(1-\pi_{0}\right)}{n}}}$$

The test statistic may be greater than the standard normal distribution $z_{2\alpha }$ for upper tailed hypothesis. Thus, one should reject the null hypothesis and accept the alternative hypothesis. The test proportion $\pi_{0}$ was set at level 0.8. This meant that it should have been a generalized conclusion for 80% of SMEs. This level was chosen due to the findings of many researchers [92,93,94,95,96,97,98,99,100,101] that summed up to the fact that over 80% of SMEs were negatively affected by the COVID-19 pandemic. The purpose of this study was to contrast this fact and show how smart sensors supported the retention of EBITDA in more than 80% of SMEs in each analyzed country during the crisis.

The following theory was developed and tested separately for each nation:

**H_c_.**  *More than 80% of Slovak (Czech, Polish, and Hungarian) SMEs balanced earnings during the crisis.*

## 3. Results

The results assessed the nexus between smart sensors and the retention of earnings by SMEs. The sample included 4491 units from the V4 region and covered a 6-year period. 

Firstly, the occurrence of a negative trend in earnings in smart enterprises was checked during the period 2016–2021. The significance level alpha was set at level 0.05 for all the investigations. Each country of the Visegrad Four was tested in turn.

The following hypotheses were mapped to the analysis in Slovakia:

**H_0a_:**  *Smart sensors balanced earnings for Slovak SMEs. There was no trend in the period of 2016–2021.*

**H_1a_.**  *Smart sensors did not balance earnings for Slovak SMEs. There was a negative trend confirmed for the period of 2016–2021.*

**Figure 1:**
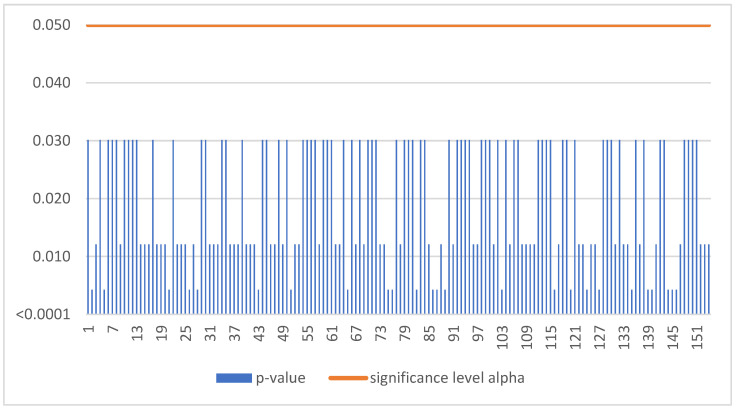
Negative trend in Slovakia.

The Mann–Kendall trend test was run for each enterprise in the sample of 1221 SMEs. If the computed p-value is lower than the significance level alpha, one should reject the null hypothesis and accept the alternative hypothesis based on Figure 1. This situation, due to the occurrence of a negative trend, was confirmed for 154 SMEs (Table 3).

**Figure 2:**
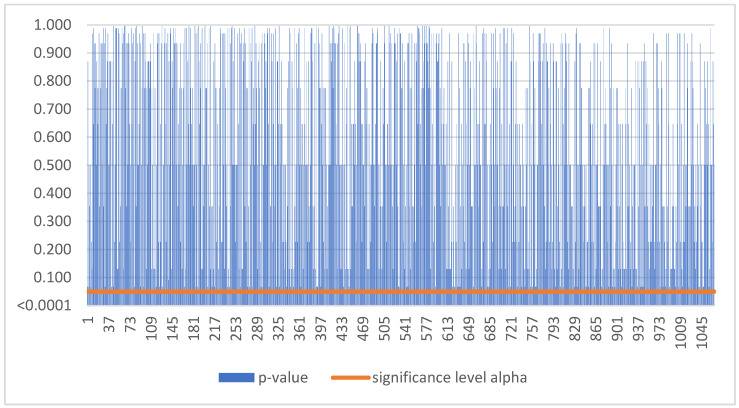
No trend in Slovakia.

Based on Figure 2, as the p-value was greater than the significance level alpha, we could not reject the null hypothesis. Thus, smart sensors balanced earnings for Slovak SMEs during the period 2016–2021 in 1067 cases (Table 3).

The following hypotheses were mapped to the analysis in Czechia:

**H_0a_.**  *Smart sensors balanced earnings for Czech SMEs. There was no trend in the period of 2016–2021.*

**H_2a_.**  *Smart sensors did not balance earnings for Czech SMEs. There was a negative trend confirmed for the period of 2016–2021.*

**Figure 3:**
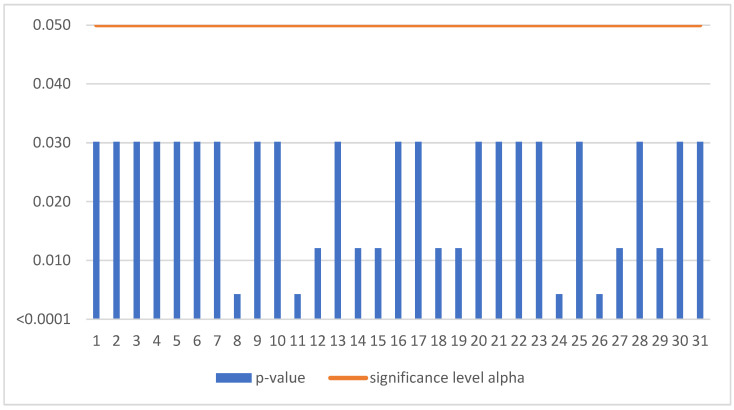
Negative trend in Czechia.

The Mann–Kendall trend test was run for each enterprise in the sample of 259 SMEs. If the computed *p*-value is lower than the significance level alpha, one should reject the null hypothesis and accept the alternative hypothesis based on Figure 3. This situation, due to the occurrence of a negative trend, was confirmed for 31 SMEs (Table 3).

**Figure 4:**
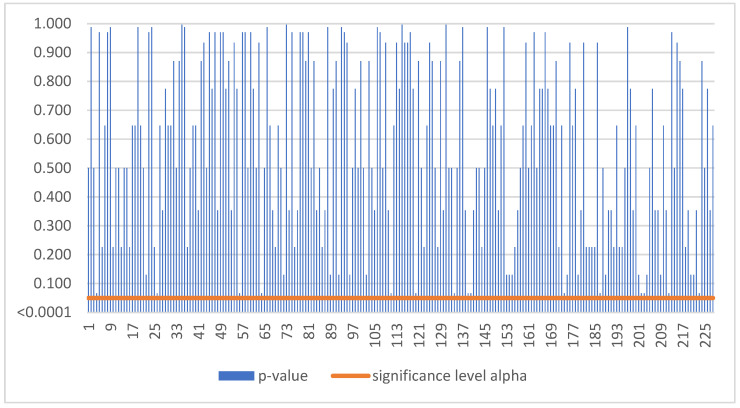
No trend in Czechia.

Based on Figure 4, as the p-value was greater than the significance level alpha, we could not reject the null hypothesis. Thus, smart sensors balanced earnings for Czech SMEs during the period 2016–2021 in 228 cases (Table 3).

The following hypotheses were mapped to the analysis in Poland:

**H_0a_.**  *Smart sensors balanced earnings for Polish SMEs. There was no trend in the period of 2016–2021.*

**H_3a_.**  *Smart sensors did not balance earnings for Polish SMEs. There was a negative trend confirmed for the period of 2016–2021.*

**Figure 5:**
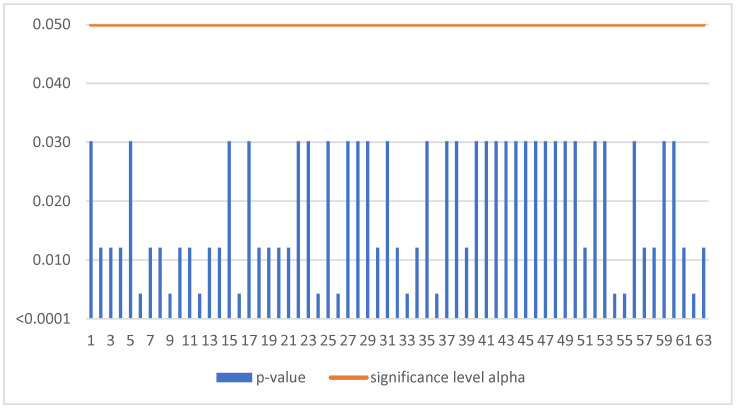
Negative trend in Poland.

The Mann–Kendall trend test was run for each enterprise in the sample of 855 SMEs. If the computed *p*-value is lower than the significance level alpha, one should reject the null hypothesis and accept the alternative hypothesis based on Figure 5. This situation, due to the occurrence of a negative trend, was confirmed for 63 SMEs (Table 3).

**Figure 6:**
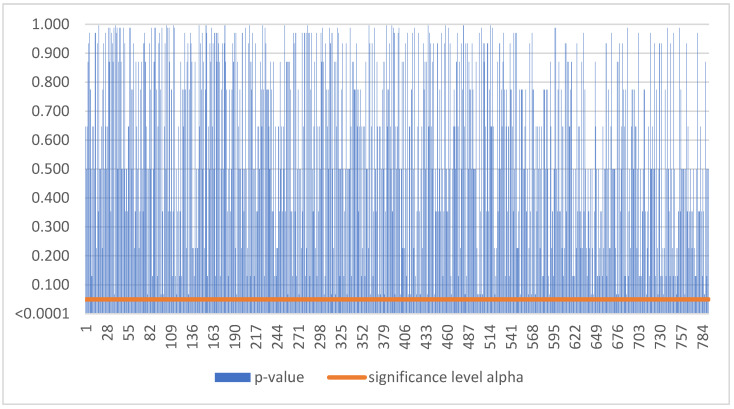
No trend in Poland.

Based on Figure 6, as the p-value was greater than the significance level alpha, we could not reject the null hypothesis. Thus, smart sensors balanced earnings for Polish SMEs during the period 2016–2021 in 792 cases (Table 3).

The following hypotheses were mapped to the analysis in Hungary:

**H_0a_.**  *Smart sensors balanced earnings for Hungarian SMEs. There was no trend in the period of 2016–2021.*

**H_4a_.**  *Smart sensors did not balance earnings for Hungarian SMEs. There was a negative trend confirmed for the period of 2016–2021.*

**Figure 7:**
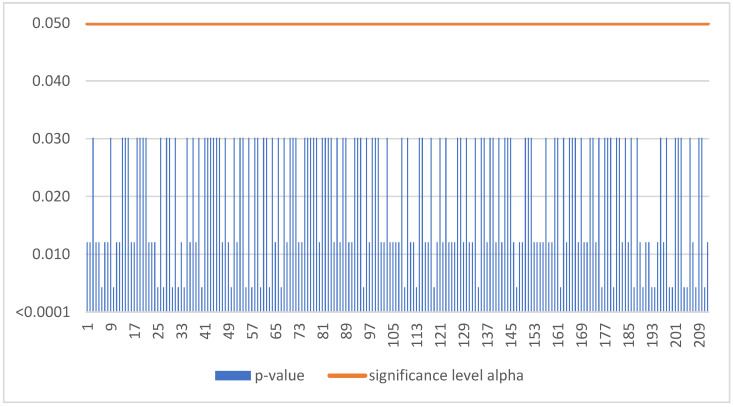
Negative trend in Hungary.

The Mann–Kendall trend test was run for each enterprise in the sample of 2156 SMEs. If the computed *p*-value is lower than the significance level alpha, one should reject the null hypothesis and accept the alternative hypothesis based on Figure 7. This situation, due to the occurrence of a negative trend, was confirmed for 212 SMEs (Table 3). 

**Figure 8:**
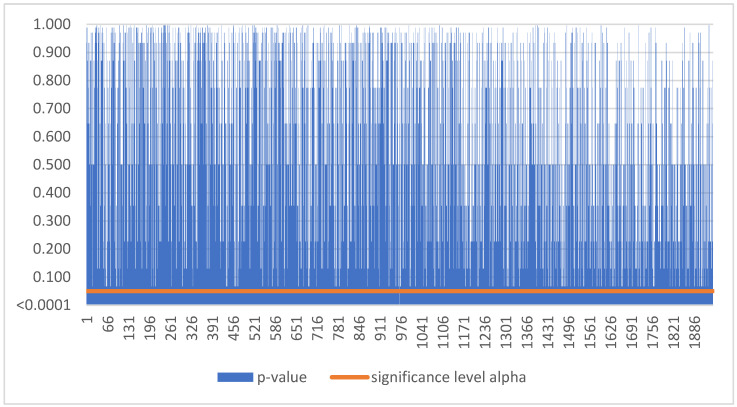
No trend in Hungary.

Based on Figure 8, as the p-value was greater than the significance level alpha, we could not reject the null hypothesis. Thus, smart sensors balanced earnings for Hungarian SMEs during the period 2016–2021 in 1944 cases (Table 3).

Secondly, the negative trend was explored in detail for 460 SMEs. The test for heterogeneity was applied individually in every enterprise with this kind of trend. The pandemic years of 2020 and 2021 were supposed to be the cause of decreased development in the time series. The significance level alpha was set at level 0.05 for all the investigations. Each country of the Visegrad Four was tested in turn.

The following hypotheses corresponded to the analysis of Slovakia:

**H_0b_.**  *There was no phenomenon of heterogeneity in earnings for smart Slovak SMEs. The pandemic years did not cause a negative disruption in the period of 2016–2021.*

**H_1b_.**  *There was a phenomenon of heterogeneity in earnings for smart Slovak SMEs. The pandemic years caused a negative disruption in the period of 2016–2021.*

**Figure 9:**
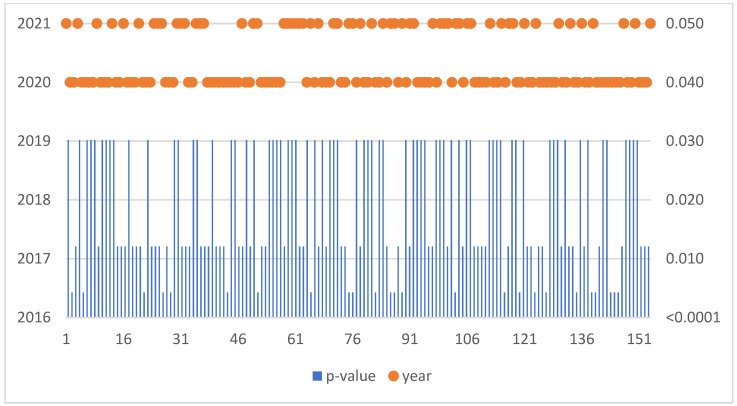
Heterogeneity in Slovakia.

Pettitt’s test was run for each enterprise in the sample of 154 Slovak SMEs. If the computed *p*-value is lower than the significance level alpha, one should reject the null hypothesis and accept the alternative hypothesis. Thus, the phenomenon of heterogeneity in earnings was accepted for each unit in the sample. Based on Figure 9, pandemic years caused negative disruption in the period 2016–2021; 2020 was a climacteric year for 95 smart SMEs, and 2021 was a critical year for 59 smart SMEs. COVID-19 was at the core of this development (Table 4).

The following hypotheses corresponded to the analysis of Czechia:

**H_0b_.**  *There was no phenomenon of heterogeneity in earnings for smart Czech SMEs. The pandemic years did not cause a negative disruption in the period of 2016–2021.*

**H_2b_.**  *There was a phenomenon of heterogeneity in earnings for smart Czech SMEs. The pandemic years caused a negative disruption in the period of 2016–2021.*

**Figure 10:**
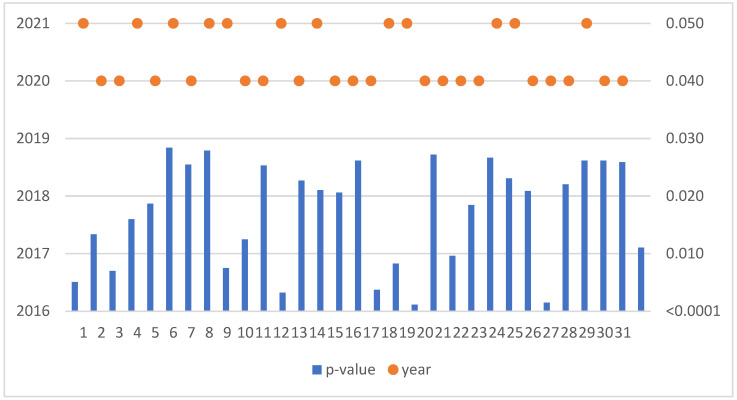
Heterogeneity in Czechia.

Pettitt’s test was run for each enterprise in the sample of 31 Czech SMEs. If the computed *p*-value is lower than the significance level alpha, one should reject the null hypothesis and accept the alternative hypothesis. Thus, the phenomenon of heterogeneity in earnings was accepted for each unit in the sample. Based on Figure 10, pandemic years caused negative disruption in the period 2016–2021; 2020 was a climacteric year for 19 smart SMEs, and 2021 was a critical year for 12 smart SMEs. COVID-19 was at the core of this development (Table 4).

The following hypotheses corresponded to the analysis of Poland:

**H_0b_.**  *There was no phenomenon of heterogeneity in earnings for smart Polish SMEs. The pandemic years did not cause a negative disruption in the period of 2016–2021.*

**H_3b_.**  *There was a phenomenon of heterogeneity in earnings for smart Polish SMEs. The pandemic years caused a negative disruption in the period of 2016–2021.*

**Figure 11:**
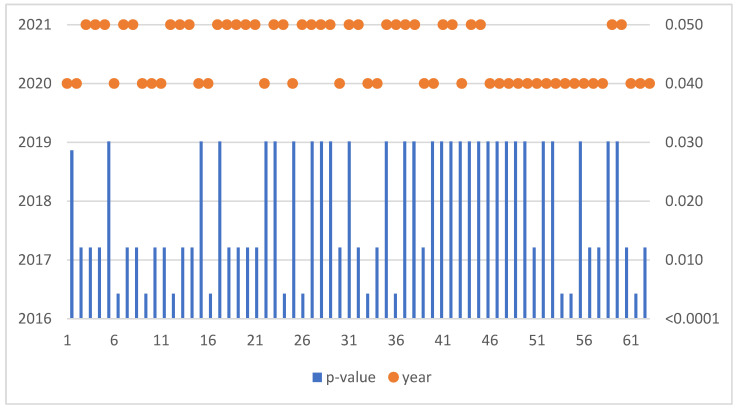
Heterogeneity in Poland.

Pettitt’s test was run for each enterprise in the sample of 63 Polish SMEs. If the computed *p*-value is lower than the significance level alpha, one should reject the null hypothesis and accept the alternative hypothesis. Thus, the phenomenon of heterogeneity in earnings was accepted for each unit in the sample. Based on Figure 11, pandemic years caused negative disruption in the period 2016–2021; 2020 was a climacteric year for 32 smart SMEs, and 2021 was a critical year for 31 smart SMEs. COVID-19 was at the core of this development (Table 4).

The following hypotheses corresponded to the analysis of Hungary:

**H_0b_.**  *There was no phenomenon of heterogeneity in earnings for smart Hungarian SMEs. The pandemic years did not cause a negative disruption in the period of 2016–2021.*

**H_4b_.**  *There was a phenomenon of heterogeneity in earnings for smart Hungarian SMEs. The pandemic years caused a negative disruption in the period of 2016–2021.*

**Figure 12:**
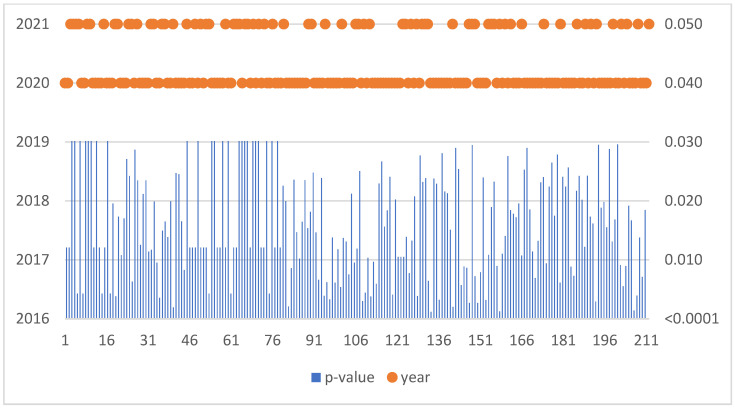
Heterogeneity in Hungary.

Pettitt’s test was run for each enterprise in the sample of 212 Hungarian SMEs. If the computed *p*-value is lower than the significance level alpha, one should reject the null hypothesis and accept the alternative hypothesis. Thus, the phenomenon of heterogeneity in earnings was accepted for each unit in the sample. Based on Figure 12, pandemic years caused negative disruption in the period 2016–2021; 2020 was a climacteric year for 143 smart SMEs, and 2021 was a critical year for 69 smart SMEs. COVID-19 was at the core of this development (Table 4).

A negative trend was proven due to the pandemic and its consequences. Finally, the study exposed the striking effect of Industry 4.0 on earnings. The tests for one proportion disclosed the percentage of SMEs that protected their earnings using smart sensors during a crisis. The findings were generalized based on the analyzed samples for the entire population. The significance level alpha was set at level 0.05 for all the investigations. Each country of the Visegrad Four was tested in turn.

The hypotheses supporting the analysis of Slovakia were:

**H_0c_.**  *80% of Slovak SMEs balanced earnings during the crisis via Industry 4.0.*

**H_1c_.**  *More than 80% of Slovak SMEs balanced earnings during the crisis via Industry 4.0.*

The z-test for comparison of one proportion tested if the difference between the proportions was equal to 0 or not. If the computed *p*-value is lower than the significance level alpha, one should reject the null hypothesis and accept the alternative hypothesis. Thus, more than 80% of Slovak SMEs balanced earnings during the crisis via Industry 4.0. This was generalized based on the *p*-value < 0.0001 from Table 5.

The hypotheses supporting the analysis in Czechia were:

**H_0c_.**  *80% of Czech SMEs balanced earnings during the crisis via Industry 4.0.*

**H_2c_.**  *More than 80% of Czech SMEs balanced earnings during the crisis via Industry 4.0.*

The z-test for comparison of one proportion tested if the difference between the proportions was equal to 0 or not. If the computed *p*-value is lower than the significance level alpha, one should reject the null hypothesis and accept the alternative hypothesis. Thus, more than 80% of Czech SMEs balanced earnings during the crisis via Industry 4.0. This was generalized based on the *p*-value = 0.0008 from Table 5.

The hypotheses supporting the analysis in Poland were:

**H_0c_.**  *80% of Polish SMEs balanced earnings during the crisis via Industry 4.0.*

**H_3c_.**  *More than 80% of Polish SMEs balanced earnings during the crisis via Industry 4.0.*

The z-test for comparison of one proportion tested if the difference between the proportions was equal to 0 or not. If the computed *p*-value is lower than the significance level alpha, one should reject the null hypothesis and accept the alternative hypothesis. Thus, more than 80% of Polish SMEs balanced earnings during the crisis via Industry 4.0. This was generalized based on the *p*-value < 0.0001 from Table 5.

The hypotheses supporting the analysis in Hungary were:

**H_0c_.**  *80% of Hungarian SMEs balanced earnings during the crisis via Industry 4.0.*

**H_4c_.**  *More than 80% of Hungarian SMEs balanced earnings during the crisis via Industry 4.0.*

The z-test for comparison of one proportion tested if the difference between the proportions was equal to 0 or not. If the computed *p*-value is lower than the significance level alpha, one should reject the null hypothesis and accept the alternative hypothesis. Thus, more than 80% of Hungarian SMEs balanced earnings during the crisis via Industry 4.0. This was generalized based on the *p*-value < 0.0001 from Table 5.

## 4. Discussion

The results were discussed to determine whether they were a close match with the last studies from the V4 region. The findings for SMEs (primarily from the transportation and automotive sectors) may be expressed through the juxtaposition of similar ones.

Gasiorek [102] did not analyze the entire V4 region; the article highlighted the skills needed for enterprises to be successful as based on responses from respondents in Poland. It is necessary to have stable earnings not only due to the technological part that was presented in this study; the study by Gasiorek [102] also reported specific soft competencies (including cognitive and social) to be imparted into the human workforce to meet the requirements of both progress in technology and the requirements of employees for Industry 4.0. Olsanova et al. [103] confirmed these conclusions in Czech conditions as well. The investigation recommended lifelong learning with technological advancements and soft skills to be smart and profitable. On the contrary, the Hungarian automotive industry does not place human resources among the success factors a priori. This was evidenced in the research by Toth-Kaszas [104].

Karmanska [105] explored the striking effect of Industry 4.0 in the transport sector. This study focused on the Internet of Things. The study used a questionnaire and an interview technique for Polish enterprises. The benefits were different because they balanced earnings. From a microeconomic view, the biggest improvements were shown in the areas of employee productivity and asset management.

Kliestik et al. [106] assessed the EBITDA of transport enterprises in the V4 region in general. There were no specifications required to be fulfilled by the enterprises. The investigation covered a period before the COVID-19 pandemic (2010–2019). The earnings were tested on an average for every year, not individually for each enterprise, as in this study. They found a downward trend in the analyzed time series that was in contrast with the provided study. In addition, its opposite effect, the effect of Industry 4.0, was particularly caused by earnings manipulation. The secondary cores were indicated as the GDP, unemployment rate, average monthly gross wage, and the ease of doing business index.

The exploration by Michalkova et al. [107] added 26 countries to the countries of V4 during the precrisis years of 2011–2019. Panel data were mapped for earnings. The study disclosed that earnings were significantly affected by the size of the enterprise. All transportation SMEs’ earnings were managed downward. This generalization of results contrasted with the ones mentioned for smart SMEs from the analyzed sample. However, very large enterprises demonstrated positive earnings. The advantage was reflected much earlier than in SMEs before the COVID-19 pandemic. This might be the consequence of the first implementation of Industry 4.0 in very large enterprises rather than in SMEs.

## 5. Conclusions

The aim of this study was to expose the striking effect of Industry 4.0 on earnings during the crisis in the Visegrad Four. Specifically, the advantages of using smart sensors for the retention of earnings as represented by EBITDA were shown. A decrease in earnings may support even the bankruptcy of SMEs. It was proven that COVID-19 caused a negative trend in earnings. During the pandemic crisis (2020–2021), however, smart sensors the balanced earnings of more than 80% of Slovak (Czech, Polish, and Hungarian) SMEs. Based on these results, Industry 4.0 and its components mean not only progress, but also the protection business in which they have been involved. This positive fact, which was based on verifiable samples, is the next reason for governments and authorities to support and speed up programs that apply smart manufacturing systems and technology.

The first limitation was that there was not an equal number of enterprises in the samples for each country. The SMEs declared the dominant use of smart sensors as a possible progression of tools. However, we did not check whether there were positive effects of other parts of Industry 4.0 that were implemented in SMEs. Further, the orientation was for all sectors and only SMEs. Thus, future research should be extended to the investigation of large and very large enterprises. In addition, the orientation per sector included balanced samples that involved Industry 4.0. The other parts of Industry 4.0 may also be tested while their effects are being examined.

## Figures and Tables

**Table 1:** Summary of last incentives from literature review.

Area Solved by Smart Sensors	Study	Reference
Sensor networks	Blake and Frajtova Michalikova (2021)	[38]
	Nagy and Lazaroiu (2022)	[39]
	Tucker (2021)	[40]
	Suler et al. (2021)	[41]
	Adams and Krulicky (2021)	[56]
Smart processes	Marinov et al. (2022)	[44]
	Blake et al. (2021)	[45]
	Griffin and Krastev (2021)	[46]
	Jiang and Qiu (2022)	[47]
	Shirmohammadli and Bahreyni (2021)	[61]
Big data	Clayton and Kral (2021)	[68]
	Hopkins and Siekelova (2021)	[69]
	Gibson (2021)	[70]
	Dawson (2021)	[71]
	Aliahmadi et al. (2022)	[72]
Smart manufacturing	Valaskova et al. (2022)	[48]
	Bhargava et al. (2022)	[49]
	Cheema et al. (2022)	[53]
	Novak et al. (2021)	[54]
	Adams and Krulicky (2021)	[55]
Vehicles	Welch (2021)	[62]
	Nandutu et al. (2022)	[63]
	Mitchell (2021)	[64]
	Aldridge and Stehel (2021)	[65]
	Shen et al. (2022)	[66]
	Lewis (2021)	[67]
AI	Ullo and Sinha (2021)	[50]
	Zhang and Wei (2020)	[51]
	Nica and Stehel (2021)	[52]
Smart governance	Cohen (2021)	[57]
	Horak (2021)	[58]
	Fonseca et al. (2021)	[59]
Smart cities	Townsend (2021)	[42]
	Harris (2021)	[43]
	Chapman (2021)	[60]

**Table 3:** Trends of SMEs.

Number of SMEs	Slovakia	Czechia	Poland	Hungary
Used sample	1221	259	855	2156
Negative trend	154	31	63	212
No trend	1067	228	792	1944

**Table 4:** Years of disruption for SMEs.

Number of SMEs	Slovakia	Czechia	Poland	Hungary
Occurred heterogeneity	154	31	63	212
Year 2020	95	19	32	143
Year 2021	59	12	31	69

**Table 5:** The z-test for comparison of one proportion for SMEs.

z-Test	Slovakia	Czechia	Poland	Hungary
Frequency (balanced earnings)	1067	228	792	1944
Sample size	1221	259	855	2156
Test proportion	0.8	0.8	0.8	0.8
Proportion	0.8739	0.8803	0.9263	0.9017
Assumption	Confirmed	Confirmed	Confirmed	Confirmed
Hypothesized difference	0	0	0	0
Difference	0.0739	0.0803	0.1263	0.1017
z (Observed value)	6.4176	3.1535	9.1911	11.7751
z (Critical value)	1.6449	1.6449	1.6449	1.6449
alpha	0.05	0.05	0.05	0.05
*p*-Value (upper-tailed)	<0.0001	0.0008	<0.0001	<0.0001

## Data Availability

The data presented in this study are available upon request from the corresponding author.
